# Engineering Melon Plants with Improved Fruit Shelf Life Using the TILLING Approach

**DOI:** 10.1371/journal.pone.0015776

**Published:** 2010-12-30

**Authors:** Fatima Dahmani-Mardas, Christelle Troadec, Adnane Boualem, Sylvie Lévêque, Abdullah A. Alsadon, Abdullah A. Aldoss, Catherine Dogimont, Abdelhafid Bendahmane

**Affiliations:** 1 Unité de Recherche en Génomique Végétale, UMR1165 ERL8196 INRA-UEVE-CNRS, Evry, France; 2 Unité de Génétique et Amélioration des Fruits et Légumes, INRA UR1052, Montfavet, France; 3 Department of Plant Production, College of Food and Agricultural Sciences, King Saud University, Riyadh, Saudi Arabia; Ecole Normale Superieure, France

## Abstract

**Background:**

Fruit ripening and softening are key traits that have an effect on food supply, fruit nutritional value and consequently, human health. Since ethylene induces ripening of climacteric fruit, it is one of the main targets to control fruit over ripening that leads to fruit softening and deterioration. The characterization of the ethylene pathway in Arabidopsis and tomato identified key genes that control fruit ripening.

**Methodology/Principal Findings:**

To engineer melon fruit with improved shelf-life, we conducted a translational research experiment. We set up a TILLING platform in a monoecious and climacteric melon line, cloned genes that control ethylene production and screened for induced mutations that lead to fruits with enhanced shelf life. Two missense mutations, L124F and G194D, of the ethylene biosynthetic enzyme, ACC oxidase 1, were identified and the mutant plants were characterized with respect to fruit maturation. The L124F mutation is a conservative mutation occurring away from the enzyme active site and thus was predicted to not affect ethylene production and thus fruit ripening. In contrast, G194D modification occurs in a highly conserved amino acid position predicted, by crystallographic analysis, to affect the enzymatic activity. Phenotypic analysis of the G194D mutant fruit showed complete delayed ripening and yellowing with improved shelf life and, as predicted, the L124F mutation did not have an effect.

**Conclusions/Significance:**

We constructed a mutant collection of 4023 melon M2 families. Based on the TILLING of 11 genes, we calculated the overall mutation rate of one mutation every 573 kb and identified 8 alleles per tilled kilobase. We also identified a TILLING mutant with enhanced fruit shelf life. This work demonstrates the effectiveness of TILLING as a reverse genetics tool to improve crop species. As cucurbits are model species in different areas of plant biology, we anticipate that the developed tool will be widely exploited by the scientific community.

## Introduction

Melon (*Cucumis melo*) belongs to the *Cucurbitaceae* family that contains about 800 species mainly distributed in tropical and subtropical regions [1]. Cucurbitaceae includes several other economically important cultivated plants, such as cucumber (*C. sativus*), watermelon (*Citrullus lanatus*), squash and pumpkin (*Cucurbita* spp.). Aside tomato (*Solanum lycopersicum*), watermelons, cucumbers and melons are the most cultivated vegetable species (http://faostat.fao.org). For decades, melon has played key roles in the field of plant molecular biology and plant physiology, serving as an excellent model organism for investigating sex determination [2], [3] and ripening processes [4], [5]. Melon is also a reference model to investigate vascular fluxes, as xylem and phloem saps can be readily collected [6], [7].

Melon is a diploid species (2n = 2x = 24) with an estimated genome size of 450 Mb [8]. Research efforts have been undertaken in order to gather genetic and genomic informations on melon, such as EST sequences [9], BAC libraries [10], [11], oligo-based microarrays [12] and high resolution genetic maps [13]–[15]. Melon has truly entered in the genomic era by the sequencing of the highly syntenic cucumber genome [16]. With the completion of the melon genome sequencing project in the near future [17], a major challenge is to determine gene functions. In plants, the most common techniques to produce altered or loss of function mutations are T-DNA or transposon insertional mutagenesis [18] and RNA interference [19]. However, because these methods are mainly based on the ability of a given plant to be transformed, their usefulness as general reverse genetics methods is limited to very few plant species. On the other hand, ethyl methanesulfonate (EMS) mutagenesis is a simple method to saturate a genome with mutations [20]. TILLING (Targeting Induced Local Lesions IN Genomes) combines advantages of random chemical mutagenesis and high throughput mutation discovery methods [21] and generates allelic series of the targeted genes which makes it possible to dissect the function of the protein as well as to investigate the role of lethal genes. This technique has been successfully applied to a large variety of organisms including plants and animals [20], [22]–[33].

To establish a reverse genetics platform in melon and to identify novel alleles of agronomic importance, we have set up a melon TILLING platform and performed a screen for mutations in genes that control fruit ripening. Fruit ripening is a process characterized by a number of biochemical and physiological processes that alter fruit firmness, colour, flavour, aroma, and texture [34], [35]. Ripening patterns are conserved across fleshy fruit species. Characterizations of homologous genes involved in fruit ripening in different species suggest that genetic mechanisms are also conserved [36]. In the fleshy fruit model, tomato, the plant hormone, ethylene, plays a central role in ripening [37]–[40]. Several fruit ripening mutants have been characterized in tomato [41]. The *rin* (*ripening-inhibitor*), *nor* (*non-ripening*) and *cnr* (*colorless non-ripening*) genes were shown to act upstream the ethylene signaling in ripening [41]–[43]. Mutations in the ethylene receptor, the *Nr* (*Never-ripe*) gene, were also shown to affect fruit ripening [44]. Ethylene biosynthesis requires the conversion of aminocyclopropane-1-carboxylic acid (ACC) to ethylene by the ACC oxidase (ACO). ACC oxidases are encoded by multigene families in plants [45], [46] and have been described to be involved in ripening, growth, and development. In melon, *CmACO1* silencing inhibits fruit ripening and extends fruit shelf life [47].

Melon is an attractive model for investigating fruit ripening. Unlike tomato, melon has two different ripening patterns, climacteric and non climacteric. To investigate further the role of ethylene in melon fruit ripening, we have developed a reference EMS mutant population under controlled conditions and established a TILLING platform. Then, we screened for mutations in 11 genes, mainly involved in ripening processes, and characterized *CmACO1* TILLING mutants. This work yields a missense mutation in *CmACO1* that inhibits fruit ripening and extends fruit storage life. The use of TILLING as a translational research tool is discussed.

## Results

### Production of CharMono mutant collection

The melon inbred line CharMono is a monoecious climacteric Charentais type cultivar (*Cucumis melo* L. subsp. *melo* var *cantalupensis*) that bears male flowers on the main stem and female flowers on axillary branches. The success of the TILLING approach relies on the construction of high quality mutant libraries. Ideally the mutant population must produce a mutation frequency that is conducive to high-throughput screening but is below a threshold that causes extensive sterility and plant development alteration. To optimize the mutagenesis, we first conducted a “kill-curve” analysis on batches of 100 seeds, using a dose range from 1% to 3% EMS (Table 1). Most M1 treated plants exhibited growth retardation at early seedling stage, but all of them recovered, except for the 3% EMS fraction for which only 40 plants were recovered. The M1 plants were assessed for seed production and seed germination. Low seed production was observed at 3% EMS with 37% of the plants producing less than 40 seeds per fruit. At 2% EMS, only 9% of the plants yielded less than 40 seeds per fruit (Table 1). Thus, the highest EMS doses allowing good seed set and seed germination, 1.5% and 2%, were retained and tested on large batches of seeds. The EMS treated seeds were sown in soil and seedlings were grown to fruit maturity in insect-proof plastic tunnels to avoid cross-pollination. Female flowers were hand pollinated with male flowers from the same plants, bagged and the fruits left to develop to maturity. M2 seeds were harvested from individual M1 plants. We also produced M2 seeds from 617 and 40 M1 plants treated with 1% and 3% EMS, respectively. In total, 4260 M2 seed stocks were collected.

**Table 1:** Impact of EMS concentration on M2 seed setting and germination.

EMS dose	M1 plants	Fruits with less than 40 seeds (%)	Seed germination (%)
**1%**	100	0	99
**1.50%**	100	1	99
**2%**	100	9	98
**3%**	40	37	97

**Figure 1:**
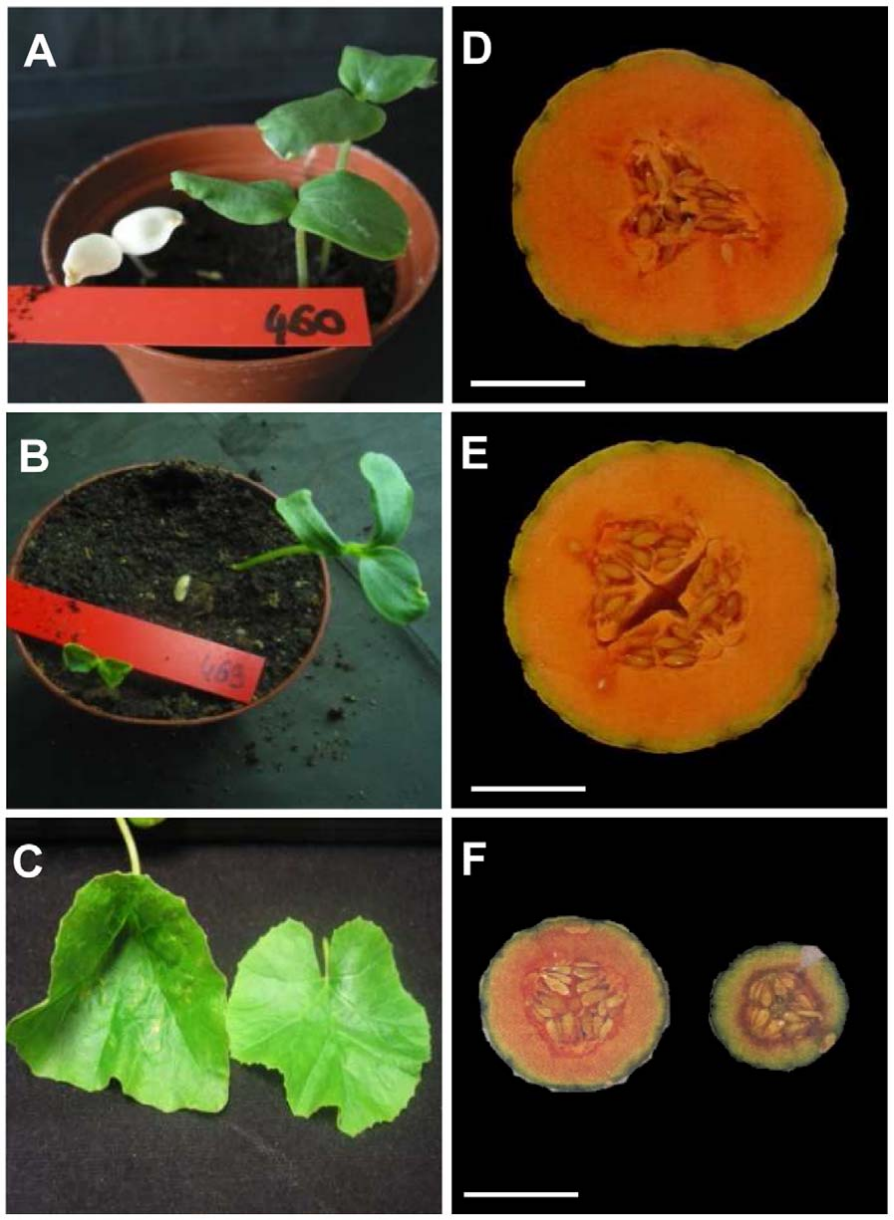
Examples of mutant phenotypes observed in CharMono mutant collection. Plant affected in cotyledon color (A) or number (B), leaf morphology (C), carpel number (E), reduced fruit size (left fruit in F) and fleshless fruit (right fruit in F). Fruit from control wild type plant is shown in (D). Scale bars: 5 cm.

To assess the quality of the mutagenesis, we investigated the rate of appearance of depigmentation mutants at the cotyledon stage and developmental alterations at fruit maturity. Albino and chlorotic plants, the most frequently observed phenotype in mutagenized populations [22], [48], occurred at the rate of 1,3% of the M2 families. This rate is similar to the rate reported for other well characterized mutant collections [49]–[51]. The most commonly observed developmental phenotypes were related to cotyledon number and morphology, dwarfism, shoot branching and plant architecture. At the flowering stage, we observed mutants with flower sex type transition, abnormal flower morphology and architecture. At the fruiting stage, altered fruit shape and size, ovary number and flesh color and thickness variations were also observed. Examples of the observed phenotypes are shown in Figure 1.

### Melon TILLING platform

To set up the TILLING platform, DNA samples were prepared from 4023 M2 families, each representing an independent M1 family and organized in pools of 8 families as described previously [25]. One key factor in TILLING is the availability of an annotated genomic sequence of the gene to be tilled, which is facilitated by the ongoing sequencing of melon genome (International Cucurbit Genomics Initiative, www.icugi.org), the availability of a large melon EST collection [9] and the 95% sequence similarity over coding regions between melon and the available cucumber genome sequence [16]. Primer design and PCR amplifications were performed as described previously [22]. Mutations were detected, in the amplified targets, using the mismatch-specific endonuclease ENDO1 [21]. Individual mutant lines were identified following a pool deconvolution step, and the mutated bases were identified by sequencing.

To assess the quality of CharMono mutant collection and to estimate the mutation density, we screened for induced mutations in 11 genes related to fruit quality. In total, we identified and confirmed by sequencing 134 induced mutations in 18,3 kb total length of tilled amplicons (Table 2). Most induced mutations were as expected, G/C to A/T transitions [20], with the exception of the three following mutations, G/C to C/G, T/A to A/T and C/G to A/T (Table S1), which have also been reported in other TILLING studies [24], [26], [52], [53].

**Table 2:** Tilled genes and mutation frequency in CharMono mutant population.

Targets	Function	Amplicon size (pb)	GC content (%)	Identified mutants	Screened M2 families	Mutation frequency
*CmDET1*	light signaling pathway	3193	35.11	24	2538	1/337 kb
*CmDHS*	eIF5A activation	2434	36.48	19	2538	1/325 kb
*CmACO1*	ethylene biosynthesis	1469	40.16	7	3306	1/694 kb
*CmNOR*	fruit ripening process	1272	39.70	9	3306	1/467 kb
*CmEXP1*	cell-wall modification	1168	46.23	11	3306	1/351 kb
*CmSP1*	Inflorescence development	1055	33.93	3	4023	1/1415 kb
*CmTCTP*	cell growth	1512	36.24	11	4023	1/553 kb
*CmCNR*	fruit ripening process	1333	40.36	11	4023	1/487 kb
*CmSGR*	chlorophyll degradation	1383	37.24	13	4023	1/428 kb
*CmACS7*	sex determinism	1680	38.57	8	4023	1/845 kb
*CmWIP1*	sex determinism	1812	31.95	18	4023	1/405 kb
	**Total**	**18311**	**37.82**	**134**	**-**	**1/573 kb**

The size of the tilled amplicons, the GC content, the number of induced alleles and the mutation frequency per amplicon are shown. The average mutation frequency in the collection is estimated to one mutation per 573 kb and is calculated as described previously [20].

Sequence analysis of the exonic induced mutations showed that 31,3% were silent, 65,1% were missense, 2,4% were stop and 1,2% were splice junction mutations (Figure S1, Table 3). The number of stop mutations was lower than the CODDLE predicted proportions. In contrast, silent, missense, and splice junction mutations were recovered in an equivalent proportion as predicted (Table 3). As many tilled amplicons harbored non coding segments, some recovered mutations could potentially affect the splicing or the stability of the mRNA, such impacts are unpredictable. Thus, non coding mutations were not characterized further. In contrast, the many non-synonymous mutations discovered were investigated to dissect the function of the proteins as they may lead to gain- or loss-of-function phenotypes. We estimated the mutation density based on Greene *et al*. [20] to 1 mutation every 573 kb. This observed density is proportional to the EMS dose used, except for the 1.5% EMS fraction showing a lower mutation frequency may be due to some experimental variations (Table 4).

**Table 3:** Expected and observed frequencies of induced mutation types in tilled gene-coding regions.

	missense	nonsense	splicing	silent
expected	64.0%	5.0%	1.7%	29.3%
observed	65.1%	2.4%	1.2%	31.3%

The percentage of the expected mutations were calculated using the CODDLE program as described previously [22].

**Table 4:** Effect of EMS dose on mutation frequency in CharMono mutant collection.

EMS dose	M1 plants	Induced mutations	Mutation frequency
**1%**	617	19	1/588 kb
**1.50%**	2130	45	1/848 kb
**2%**	1473	67	1/356 kb
**3%**	40	3	1/146 kb
**Total**	**4260**	**134**	**1/573 kb**

### Characterization of *CmACO1* TILLING mutants

**Figure 2:**
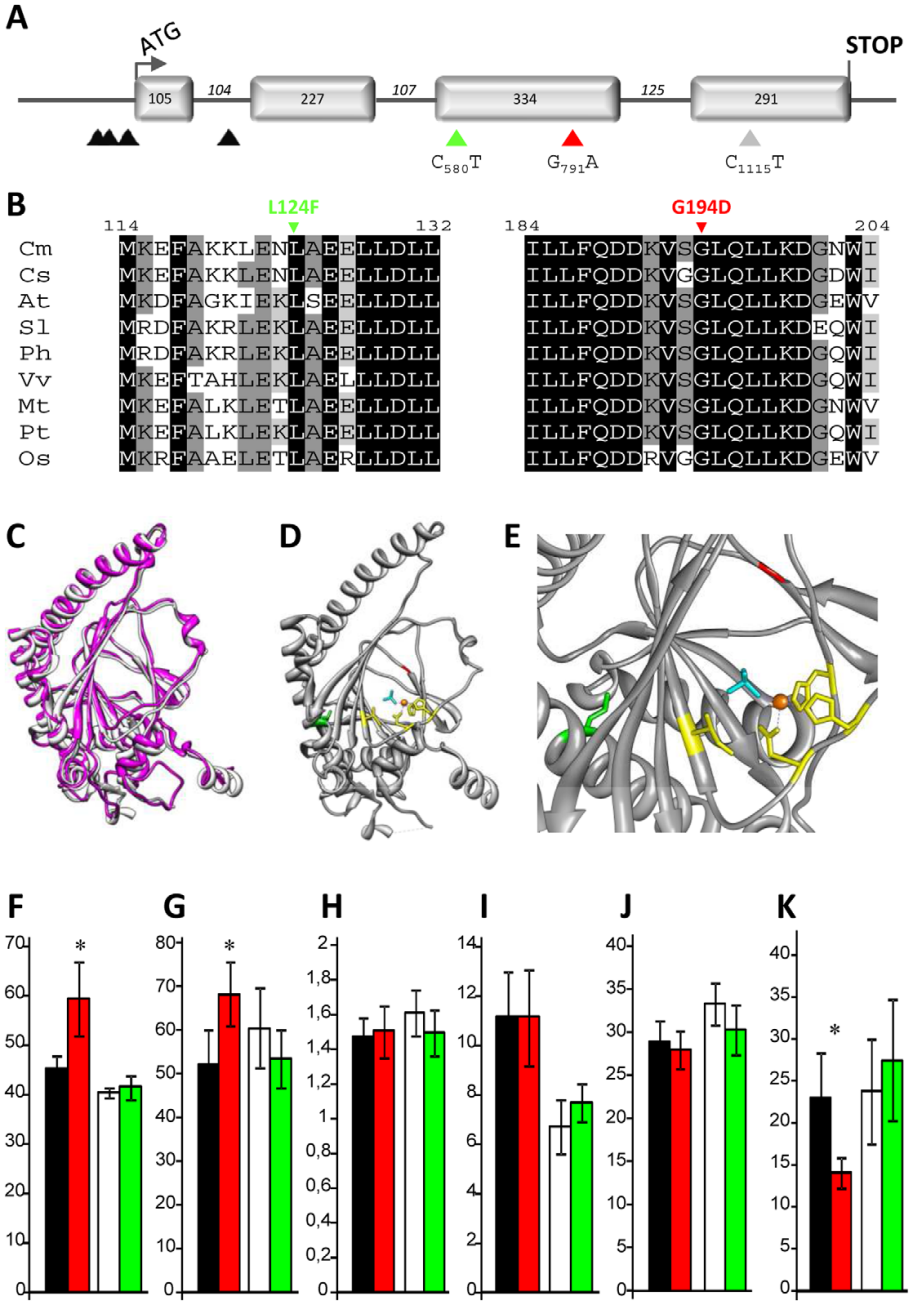
Sequence and structural analysis of *CmACO1.* (A) Schematic diagram of *CmACO1* gene structure. The numbers indicate the size of the 4 exons (filled boxes) and the 3 introns (black lines) in bps. Intronic, silent and the L124F and G194D missense mutations are represented by black, grey, green and red triangles, respectively. (B) Amino acid alignment of CmACO1 (*Cucumis melo*, Q04644) and homologous proteins from *Cucumis sativus* (Cs, BAA33377), *Arabidopsis thaliana* (At, NP_171994), *Solanum lycopersicum* (Sl, P05116), *Petunia x hybrida* (Ph, Q08506), *Vitis vinifera* (Vv, XP_002273430), *Medicago truncatula* (Mt, AAL35971), *Populus trichocarpa* (Pt, XP_002320487) and *Oryza sativa* (Os, NP_001063330). Numbers above the alignment indicate the amino acid positions along the CmACO1 protein. L124F and G194D EMS-induced mutations are shown above the alignment in green and red, respectively. (C–D) 3D structure model of CmACO1. (C) Superposition of the Petunia ACO structure determined by X-ray crystallography [60], indicated in pink and the 3D model of CmACO1, indicated in white. The melon model was determined using the Geno3D server (http://geno3d-pbil.ibcp.fr). (D and E) Zoom in of the ACO1 active site. Yellow sticks represent Ile171, His177, Asp179 and His234 residues involved in the Fe (II) cofactor binding (orange ball). Blue sticks represent the ligating phosphate or sulfate ion. Amino acids L^124^ and G^194^ are represented in green and red sticks, respectively. (F–K) Fruit-related traits of the L124F and G194D EMS mutants. Data for the duration between the pollination and the fruit ripening (days, F), firmness (Durofel index, G), shape (ratio length/width in mm, H), soluble sugars (° Brix, I) and flesh (J) and rind (K) color (b value) are shown. G124D mutant and its relative wild type control and L124F and its relative wild type control are indicated by red, black, green and open bars, respectively. Asterisks indicate significant differences.

Fruit softening is a major factor that determines fruit quality and shelf life. In melon and tomato, silencing of enzymes or regulators of the ethylene biosynthesis pathway inhibits fruit ripening and extend fruit storage life [47], [54]–[58]. To identify melon lines with improved fruit shelf life, we screened for mutations in the CmACO1 enzyme that catalyses the last step of ethylene biosynthesis (Figure 2A). In this TILLING screen, we identified seven independent point mutations among which two led to L124F and G194D missense mutations. The L124F and G194D changes occurred in a highly conserved amino acid positions and may therefore affect the function of the protein (Figure 2B, [59]). However, X-ray crystallography studies determined that L^124^ is located in the α-5 helix of the protein away from the active site and thus, it is predicted to not affect the function of the protein (Figure 2C–E, amino acid indicated in green; [60]). In contrast, the residue G^194^ is located in the β-7 strand, one of the eight β strands (β-4 to β-11) that form the distorted double-stranded β helix (DSBH or jellyroll) core of the active site, common to all members of the 2-OG-oxygenases [60]–[65]. The residue G^194^ is located in the inner face of the active site, and thus, a mutation at this position is predicted to affect the function of the protein (Figure 2C-E, amino acid indicated in red; [60]).

To test whether induced mutations in *CmACO1* could affect melon fruit ripening, L124F and G194D TILLING mutants were evaluated for different fruit traits (Figure 2F–K). The L124F mutant did not show any difference from its wild type control for the evaluated fruit traits. This is consistent with the conservative L124F mutation, two amino acids with hydrophobic side chains, and the position of the L^124^ residue away from the active site of the protein (Figure 2F–K, white and green bars). In contrast, the duration between the pollination and the fruit ripening as well as the fruit firmness were significantly increased in the G194D mutant compared to the wild type control (Figure 2F and G, black and red bars). G194D mutation was also associated with a significant change in the fruit rind color leading to a delayed fruit yellowing (Figure 2K). Fruit shape, soluble sugar content and flesh color showed no differences between the G194D mutant line and the wild type control (Figure 2H–J). No differences were also observed for the fruit peduncle abscission layer that is activated at fruit maturity and lead the fruit to drop from the plant.

To confirm the observed phenotypes, G194D heterozygote mutant lines were selfed and 40 homozygote mutant and wild type segregants were assessed for the above fruit traits. Homozygous wild type plants for this locus had no alteration of any fruit traits described above and behaved as the CharMono wild type parent. As predicted, plants homozygous for the G194D mutation presented an increased duration between the pollination and the fruit ripening, a better fruit firmness and a delayed fruit yellowing without any changes in fruit shape, peduncle abscission layer, soluble sugar content and flesh color. These data confirmed that the observed phenotypes resulted from G194D induced mutation in *CmACO1*. To investigate the robustness of the observed phenotypes, we repeated the phenotypic characterization of the mutant lines in two different locations, Montfavet and Saint Rémy de Provence, and confirmed the observed phenotypes (Figure S2).

## Discussion

In the genomic era and in the absence of efficient tools for homologous recombination, TILLING has become an obligate technology to dissect gene function as well as to engineer alleles of agronomic importance in crops. To set up the melon TILLING platform, we first developed a reference EMS mutant collection under controlled conditions. To assess the quality of the mutagenesis, we estimated the rate of occurrence of chlorotic and albino phenotypes. The observed frequency of 1,3%, comparable to well characterized mutant collections, confirmed the quality of the mutagenesis [49]–[51]. Genomic DNA was prepared from the mutant lines and organized in pools for bulked screening using the mismatch specific endonuclease, Endo1 [21]. The value of this melon mutant collection was then confirmed by screening for mutation in 11 genes. On average, we identified 8 alleles per tilled kilobase, calculated from the tilled 18,5 kb and the 134 identified alleles. We also estimated the overall mutation rate as one mutation every 573 kb in our CharMono mutant collection. This mutation frequency is two fold lower than the rate of one mutation per 200 kb reported for *Pisum sativum* [22], two fold higher than the rate of one mutation per megabase reported for barley [53] and equivalent to the rate of mutations described in tomato [24], [25]. A much more saturated mutation density has been observed in polyploid species which withstand much higher doses of EMS without obvious impact on plant survival (1/40 kb in tetraploid wheat and 1/24 kb in hexaploid wheat [66]).

Fruit ripening and softening are key traits that determine the shelf life of fleshy climacteric fruits. Post-harvest losses can reach 70% in developing countries because of the lack of post-harvest infrastructure to store and to retail the commodities. A wide list of approaches, ranging from the application of growth regulators to delay ripening, fruit storage in controlled atmospheres and breeding or genetic engineering of leader lines have been used to control fruit ripening and softening. Ethylene has been identified as the major hormone that controls fruit ripening and softening in climacteric fruits. Investigations in the model species as well as in crops have accumulated considerable evidences at the genetic, physiological, biochemical and molecular levels that pointed out the ethylene function at various levels. This includes ethylene biosynthesis, its perception by the target cells through specific receptors, signal transduction pathway involving both positive and negative regulators and finally regulation of target genes expression by transcription factors such as ethylene response factors [46]. The final step of ethylene biosynthesis in plants is catalyzed by the ACC oxidase which converts ACC to ethylene. ACC oxidases belong to a multigene family and members have been shown to be highly expressed during fruit ripening in climacteric fruits. ACC oxidase role in ripening was demonstrated by co-suppression of the fruit ACC oxidases in fleshy fruit plants. The ACC oxidases silenced fruits exhibited non-softening phenotypes and thus, an extended shelf life [47], [54]–[58].

The TILLING of *CmACO1* identified a mutation at the conserved G^194^ residue, located in the active site of the enzyme. Detailed phenotypic characterization of the TILLING mutants showed that the G194D mutation mimic the phenotype of *CmACO1* antisense line [47]. The G194D mutant line showed longer fruit maturation, enhanced fruit firmness and delayed fruit yellowing. As reported for the antisense ACC oxidase melon lines [47], there are also no difference in the fruit sugar content, shape or flesh color. Besides, we noticed that the peduncle abscission layer that is activated at fruit maturity in wild type plant was also observed on the G194D mutant fruits in contrast with the antisense melon lines that failed to drop even at a very late developmental stage (65 days) [47]. This difference could be explained by the antisense strategy that can not, with complete conviction, exclude that other related homologous enzymes are affected in the peduncle abscission layer.

In conclusion, to investigate fruit maturation and fruit softening we tilled a list of candidate genes and isolated key mutants that enhanced melon fruit quality (data not shown). Through the TILLING approach we knocked out a specific gene, *CmACO1* and pointed out an important amino acid, Glycine at the position 194, for the fruit maturation, not reported previously. Moreover, we created a new allele for the melon post-harvest management (maturation duration) and transport (firmness), which is an economically attractive trait for breeders. This is even more important regarding the transgenic strategies that face strong concern in public acceptance. The characterization of the other mutants will likely deliver alleles that may be exploited in melon breeding. Cucurbits are also an important model species in many key areas of plant research, including sex determination [2], [3], [67], fruit maturation [4], [5] and the investigation of vascular trafficking of molecules [6], [7]. Hence, by opening the TILLING platform to the scientific community, we hope to fulfill the expectations of both crop breeders and scientists who are using melon as their model of study.

## Materials and Methods

### Plant material and EMS mutagenesis

Experiments were carried out using the melon inbred line CharMono, a monoecious climacteric Charentais type cultivar (*Cucumis melo* L. subsp. *melo* var *cantalupensis*). Mature seeds from CharMono line were immersed in bottles containing 250 ml of EMS diluted at right concentration in deionized water, and placed on a rotary shaker overnight (16 h) at 24°C in the dark. The EMS treatment was stopped by addition of 100 ml of Na_2_SO_3_ at 0,1 M. Treated seeds were then transferred into a new solution of Na_2_SO_3_ 0,1M for 15 minutes. The EMS solution was then removed and seeds were washed extensively. Treated seeds were sown in soil and grown under insect-proof plastic tunnels according to standard melon agronomic practice. At the end of the fruit ripening phase, M2 seeds were collected from individual M1 plants and stored.

### Genomic DNA extraction and pooling

Sixteen melon leaf discs (diameter 10 mm) from eight individual plants per M2 family were collected in 96-well plates containing 2 steel beads (4 mm) per well, and tissues were ground using a bead mill. Genomic DNA was isolated using the Dneasy 96 Plant Kit (Qiagen, Hilden, Germany). DNAs were quantified on a 1% agarose gel using λ DNA (Invitrogen, Carlsbad, USA) as a concentration reference. DNA samples were then diluted ten fold and pooled eight fold in a 96-well format.

### PCR amplification and mutation detection

PCR amplification is based on nested-PCR. The first PCR amplification is a standard PCR reaction using target-specific primers (Table S2) and 4 ng of melon genomic DNA. One µl of the first PCR served as a template for the second nested PCR amplification, using combination of specific primers carrying M13 tail and M13 universal primers, M13F700 (5′-CACGACGTTGTAAAACGAC-3′) and M13R800 (5′-GGATAACATTTCACACAGG-3′), labelled at the 5′end with infra-red dyes IRD700 and IRD800 (LI-COR®, Lincoln, Nebraska, USA), respectively. Mutation detection was carried out as described previously [22]. The identity of the mutations was determined by sequencing. TILLING request should be addressed to the corresponding author.

### Sequence analysis tools

CODDLE (Codons Optimized to Discover Deleterious Lesions, http://www.proweb.org/coddle/) was used to identify regions of the target gene in which G/C to A/T transitions are most likely to result in deleterious effects on the protein. PARSESNP (Project Aligned Related Sequences and Evaluate SNPs, http://www.proweb.org/parsesnp/) was used to illustrate the distribution of mutations within the gene, and to indicate the nature of each single mutation. SIFT (Sorting Intolerant from Tolerant, http://sift.jcvi.org/www/SIFT_seq_submit2.html) was used to predict the impact of the mutation on the protein. Multiple sequence alignment of full-length protein sequences was performed using ClustalW (http://www.ebi.ac.uk/Tools/clustalw2).

### Protein structure modeling

The CmACO1 three-dimensional structures were generated using the Geno3D server (http://geno3d-pbil.ibcp.fr). Superposition of the petunia ACO structure (1W9Y.pdb) determined by X-ray crystallography [60], and our CmACO1 model, was carried out and visualized using Chimera (http://www.cgl.ucsf.edu/chimera).

### TILLING mutant phenotyping

Mutant and wild type plants were grown in two locations, Montfavet and Saint Rémy de Provence, in France, under greenhouse conditions. Fruits were harvested at maturity, according to the harvest indicators usually observed in Charentais type fruits, namely: the development of abscission layer, yellow rind and death of the first leaf beside the peduncle. At maturity, the duration between pollination and harvesting was evaluated. Mature fruit shape was measured as the ratio of the polar diameter (peduncle to blossom end), referred to as fruit length, to the equatorial width of the fruit (measured halfway between the peduncle and blossom end). Soluble sugar content of the flesh was measured with a hand refractometer. Firmness was measured in two different areas of the flesh after cutting the fruit lengthwise, using a Durofel electronic penetrometer or with Gullimex penetrometer equipped with an 8 mm diameter probe. Rind and flesh colors were recorded using a Minolta colorimeter at two different sites per fruit. The (b) parameter was selected for monitoring changes in the yellow hue [68].

## Supporting Information

Figure S1 **Graphic representations of genes screened for mutations.** Black boxes represent the exons. Lanes linking exons indicate introns. Dashed lines in red indicate the genomic regions screened for mutations. Triangles pointing up indicate mutations in coding regions, whereas those pointing down indicate mutations in intron-exon splicing sites. Red, black and grey triangles represent alterations causing truncations, missense and silent mutations, respectively. Mutations in introns, 5′ and 3′UTR are not shown.
(TIF)

Figure S2 **Multi-location evaluation of fruit traits for the G194D EMS mutant.** (A–F) Fruit-related traits tested at INRA Avignon experimental station in Montfavet, France and (G–H) at a second experimental station at Saint Rémy de Provence, France. Data for the duration between the pollination and the fruit ripening (days, A and G), firmness (Durofel index, B and kg/0,5 cm^2^, H), shape (ratio length/width in mm, C and I), soluble sugars (° Brix, D and J) and flesh (E and K) and rind (F) color (b value) are shown. G124D mutant and its relative wild type control are indicated by red and black bars, respectively. Blue stars indicate significant difference. Values represent the means of measurements obtained for at least 10 fruits from different plants.
(TIF)

Table S1 **Missense, STOP and splicing induced mutations.** Nucleotide substitution, amino acid changes and the predicted impact on the protein function are reported for each EMS-mutant line. Asterisks indicate non conventional EMS mutations. The amino acid substitution is predicted damaging if the score is < or  = 0.05 and tolerated if the score is >0.05.
(XLS)

Table S2 **Primers used in this study.** Asterisk indicate primers labeled with infra-red dyes IRD700 (forward primers) and IRD800 (reverse primers).
(XLS)
