# Supplementary figures and images for: Engineering Melon Plants with Improved Fruit Shelf Life Using the TILLING Approach
Source: PLoS One. 2010 Dec 30;5(12):e15776. doi: 10.1371/journal.pone.0015776 (PMC3012703; doi:10.1371/journal.pone.0015776)

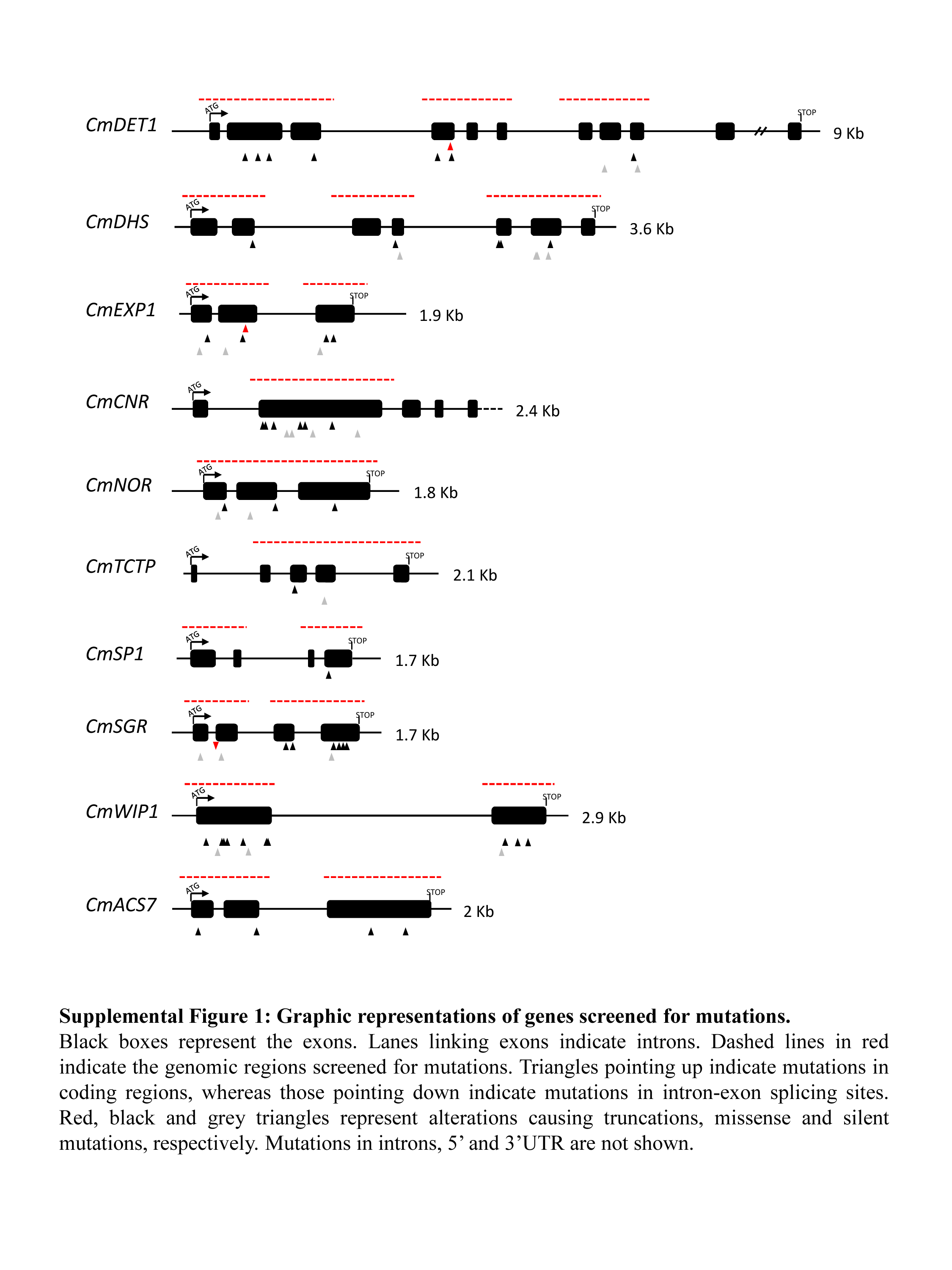

Supplement: Figure S1 — Graphic representations of genes screened for mutations. Black boxes represent the exons. Lanes linking exons indicate introns. Dashed lines in red indicate the genomic regions screened for mutations. Triangles pointing up indicate mutations in coding regions, whereas those pointing down indicate mutations in intron-exon splicing sites. Red, black and grey triangles represent alterations causing truncations, missense and silent mutations, respectively. Mutations in introns, 5′ and 3′UTR are not shown. (TIF) [file pone.0015776.s001.tif]

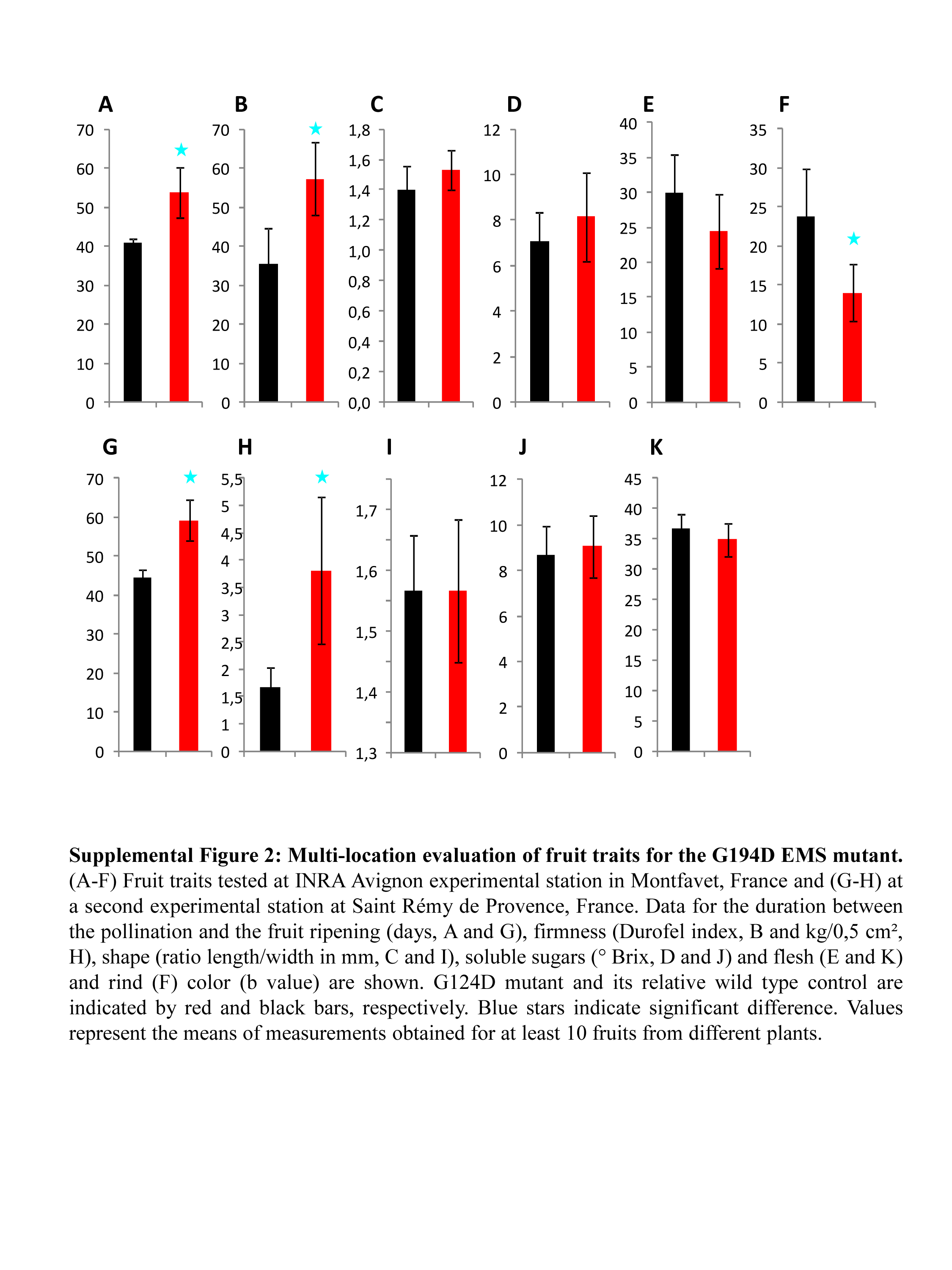

Supplement: Figure S2 — Multi-location evaluation of fruit traits for the G194D EMS mutant. (A–F) Fruit-related traits tested at INRA Avignon experimental station in Montfavet, France and (G–H) at a second experimental station at Saint Rémy de Provence, France. Data for the duration between the pollination and the fruit ripening (days, A and G), firmness (Durofel index, B and kg/0,5 cm2, H), shape (ratio length/width in mm, C and I), soluble sugars (° Brix, D and J) and flesh (E and K) and rind (F) color (b value) are shown. G124D mutant and its relative wild type control are indicated by red and black bars, respectively. Blue stars indicate significant difference. Values represent the means of measurements obtained for at least 10 fruits from different plants. (TIF) [file pone.0015776.s002.tif]
